# Protocol for the development of a core domain set for individuals with ankle osteoarthritis

**DOI:** 10.1186/s13063-022-06692-0

**Published:** 2022-09-05

**Authors:** Sultan Ayyadah Alanazi, Bill Vicenzino, Christiaan J. A. van Bergen, David J. Hunter, Erik A. Wikstrom, Hylton B. Menz, Yvonne M. Golightly, Michelle D. Smith

**Affiliations:** 1 The University of Queensland, School of Health and Rehabilitation Sciences: Physiotherapy, Brisbane, Australia; 2 Department of Physical Therapy, College of Applied Medical Sciences, Majmaah University, Al-Majmaah, 11952 Saudi Arabia; 3 Department of Orthopedic Surgery, Amphia, Breda, the Netherlands; 4 Department of Orthopedic Surgery and Sports Medicine, Erasmus University Medical Center, Rotterdam, the Netherlands; 5 Sydney Musculoskeletal Health, Kolling Institute, The University of Sydney, Sydney, Australia; 6 Rheumatology Department, Royal North Shore Hospital, Sydney, Australia; 7 MOTION Science Institute, Department of Exercise and Sport Science, University of North Carolina at Chapel Hill, Chapel Hill, NC USA; 8 Discipline of Podiatry, School of Allied Health, Human Services and Sport, La Trobe University, Melbourne, Victoria Australia; 9 College of Allied Health Professionals, University of Nebraska Medical Center, Omaha, NE USA; 10 Thurston Arthritis Research Center, University of North Carolina, Chapel Hill, NC USA

**Keywords:** Core domain set, Core outcome set, Ankle Osteoarthritis, Delphi survey, Consensus, Patient-reported outcome measures

## Abstract

**Background:**

Ankle osteoarthritis (OA) is a debilitating health condition that is increasing in prevalence. Currently, there are no evidence-based guidelines for managing ankle OA. One of the current challenges to establishing guidelines is the lack of a widely agreed-upon set of outcome measures that are consistently used in ankle OA research. Without a set of agreed-upon outcome measures, it is difficult to synthesise clinical trial outcomes through meta-analysis—an essential element of evidence-informed practice. In order to develop an appropriate set of outcome measures for ankle OA, it is important first to develop a core domain set. In this protocol, we describe the methodological approach that we will use to develop such a core domain set for ankle OA.

**Methods:**

We established an international steering committee to guide the development of a core domain set for ankle OA. The core domain set development will follow a multi-staged approach consisting of three phases, involving participation by patients and clinicians/healthcare professionals. In phase 1, a list of candidate domains will be gleaned from (a) a scoping review of outcome measures used in ankle OA research, (b) qualitative interviews with individuals with ankle OA, and (c) qualitative interviews with healthcare professionals with expertise in ankle OA. In phase 2, the steering committee will review and generate a list of candidate domains from those gleaned in phase 1. In phase 3, this list of candidate domains will be considered in a Delphi process to reach a consensus on a core domain set. We anticipated this will involve 3 rounds of surveys.

**Conclusion:**

This protocol describes the methods that will be used to develop a core domain set of health-related aspects for ankle OA. Importantly, it will include both healthcare professional and patient involvement. This is a prerequisite step to developing a core outcome set for ankle OA that should be reported in all clinical trials for ankle OA. The findings will be widely disseminated across peer-refereed publication(s) and national and international conferences, as well as via relevant professional societies, patient support group organisations, and social media platforms.

**Project registration:**

This project is registered with the Core Outcome Measures in Effectiveness Trials (COMET) database on 17 March 2021. https://www.comet-initiative.org/Studies/Details/1837.

**Supplementary Information:**

The online version contains supplementary material available at 10.1186/s13063-022-06692-0.

## Background

Ankle osteoarthritis (OA) is a debilitating health condition characterised by pain, dysfunction, impaired mobility, and poor quality of life [1–3]. People with ankle OA report similar disability to people who have end-stage hip OA [4], radiculopathy, renal failure, or congestive heart failure [5]. The reported prevalence of ankle OA in adults varies from 3.4% [6] to 6.5% [7]. Ankle OA is unique in its aetiology as it commonly occurs as a consequence of ankle injuries, such as fractures and sprains [8–10]. Due to this post-traumatic nature, many individuals with ankle OA are relatively young (in their third decade) [11] and have considerable years living with the consequences of the condition [12].

The recommended management of ankle OA is unclear. There are no evidence-informed clinical practice guidelines for ankle OA and a lack of high-quality studies of ankle OA management [13, 14]. A systematic review investigating non-surgical interventions of ankle OA could not identify any evidence-informed intervention for ankle OA [15]. Surgical interventions for ankle OA, which are often considered to be the last line of treatment, have shown limited success [16, 17]. The current state of ankle OA literature is limited by the heterogeneity of outcome measures, which leads to difficulty comparing and synthesising data in systematic reviews and meta-analyses [18–20]. The vast array of outcome measures in previous research suggests a lack of agreement on outcome measures that should be assessed in ankle OA. This lack of uniformity challenges researchers who are selecting outcomes to use when studying interventions for ankle OA and continues to impede the synthesis of data from primary studies.

The development of a core outcome set has been recommended to address issues of outcome measure inconsistency [21–23]. A core outcome set is an agreed-upon minimum set of outcomes that should be measured and reported in all clinical trials for a particular health condition [24] but does not restrict the use of additional outcomes in relation to research aims. A core outcome set has the potential to increase the reporting of relevant outcomes, reduce the risk of selective outcome reporting, and increase the likelihood of conducting meta-analyses of the highest level of evidence to inform clinical practice [24]. A survey of co-ordinating Editors of Cochrane Review Groups has shown support for the development of a core outcome set and agreement that a core outcome set would enhance the reliability of systematic reviews [25].

The Outcome Measures in Rheumatology (OMERACT) [22, 26] and Core Outcome Measures in Effectiveness Trials (COMET) [23] initiatives provide methodological guidance for developing a core outcome set. This guidance involves two essential components. First is the development of an agreed-upon set of health-related domains—a core domain set—that cover all aspects of a health condition [22, 24]. A domain is an aspect of a health condition (e.g. ankle OA) that needs to be measured to comprehensively cover the impact of the condition and to appropriately assess the effects of treatment. An agreed-upon core domain set will provide guidance on what domains should be measured in ankle OA. The second component of this process is to reach agreement on a set of core outcome measurement instruments that represent the minimum set that should be used to assess the domains in the core domain set [22, 24]. This guides how each core domain should be measured. The involvement of key stakeholders (e.g. patients, clinicians, researchers) in the development of a core domain set is deemed to be important to ensure that the core outcome set is relevant to all stakeholders involved in the health condition [22, 24, 27].

In light of the heterogeneity of outcomes in ankle OA research and lack of guidance on outcome measures selection, the aim of our research is to develop an agreed-upon core domain set for ankle OA—the first step of the core outcome set development process. We describe the methods that will be used to generate candidate domains. We also explain the methodological guidance that we will use to achieve consensus on a core domain set for ankle OA. This is in line with COMET and OMERACT guidance [22, 24].

## Scope

Individuals who have ankle OA are the focus of this core domain set—predominantly in clinical trials of treatments. The core domain set will lead to consistent use of outcome measures in ankle OA research, which will better inform clinical practice guidelines and clinical practice.

## Methods/design

This study will undertake a multi-stage process to develop a core domain set for ankle OA. It will engage an international group of individuals who have ankle OA and healthcare professionals (e.g. clinicians and researchers) with experience in the treatment of ankle OA. This protocol is devised in accordance with Core Outcome Set-STAndardised Protocol Items (COS-STAP) guidance [28]. The COS-STAP checklist of this project is provided in Additional file 1.

## Stakeholder participation

For a core domain set to be effectively implemented, there should be involvement of stakeholders in its development, including patients who live with the condition, and researchers/healthcare professionals (e.g. physiotherapists, rheumatologists, orthopaedic surgeons, outcomes researchers and statisticians) who work with individuals who have the condition [24, 27, 29, 30]. For this core domain set, we will include the following stakeholders: individuals who have ankle OA and healthcare professionals (e.g. researchers, clinicians). The COMET and the OMERACT initiatives have advocated for the involvement of patients in the development process of a core domain set [29, 31] as patients’ perspectives of living with the condition may differ from the perspectives of healthcare professionals [24, 32]. As such, several core domain developers have included patients in the development process [32–35], and their inclusion is considered to be essential.

### Participant recruitment: individuals with ankle OA

Eligibility criteria for individuals with ankle OA will be as follows: aged ≥ 18 years, ankle pain on most days of the month for the last 3 months, a radiological diagnosis of ankle OA by a healthcare professional (i.e. joint space narrowing +/− the presence of osteophytes [36]), ankle OA as their primary health problem/concern, and able to read and write in English.

Individuals with ankle OA will be recruited through healthcare professionals and using different forms of media. We will recruit via healthcare professionals involved in the study (e.g. using research or patient databases containing individuals who have consented to be contacted for future research opportunities), other healthcare professionals who see ankle OA patients, and through flyers posted in waiting rooms of clinics. The aim of the study, eligibility criteria, and benefits of participation will be introduced to potential participants. Referring healthcare professionals will screen for study eligibility.

We will also recruit individuals with ankle OA via social media (e.g. Facebook), community online/paper advertisements (e.g. Weekend Notes), and arthritis websites (e.g. Arthritis Australia, Queensland Arthritis). Ankle OA participants who are recruited via social media and community advertisements will be screened for eligibility using a three-stage screening process. First, respondents will complete an online survey that asks questions pertaining to eligibility criteria. Second, those meeting selection criteria on the online survey will be assessed over a video link by a registered sports and exercise physiotherapist (author MDS) to ensure that pain is consistent with ankle OA. Third, those meeting clinical selection criteria will be screened for radiological evidence of ankle OA. Thus, study participants will have evidence of radiological ankle OA and localised ankle joint pain as their primary health-related concern. Eligible participants will be given a QR code to provide consent and complete the study survey.

### Participant recruitment: healthcare professionals

The eligibility criteria for healthcare professionals to participate in the development of this core domain set are as follows: ≥ 18 years of age; qualified as a healthcare professional (e.g. orthopaedic surgeon, physiotherapist, athletic trainer, podiatrist, rheumatologist); and able to read and communicate in English. We will aim to recruit as many healthcare professionals as possible across a range of professions, geographical locations, and sex.

We will identify healthcare professionals who have a special interest in ankle OA and invite them to participate in this study. This will be achieved via a number of avenues—by conducting searches of authors of peer-review publications on ankle OA that were published in the last 10 years (identified from our scoping review of the literature) and approaching the following organisations: Osteoarthritis Research Society International, the International Foot and Ankle OA Consortium, the Foot and Ankle Disorder OMERACT Group, the Australian Foot and Ankle Research Network, and the International Ankle Consortium. All identified healthcare professionals will be sent an email invitation that will contain a brief background about the research and outline the purpose of the study. Interested healthcare professionals will sign an authorship agreement, stating their responsibilities in the group authorship on the manuscript reporting the consensus results.

## Consensus process

**Fig. 1:**
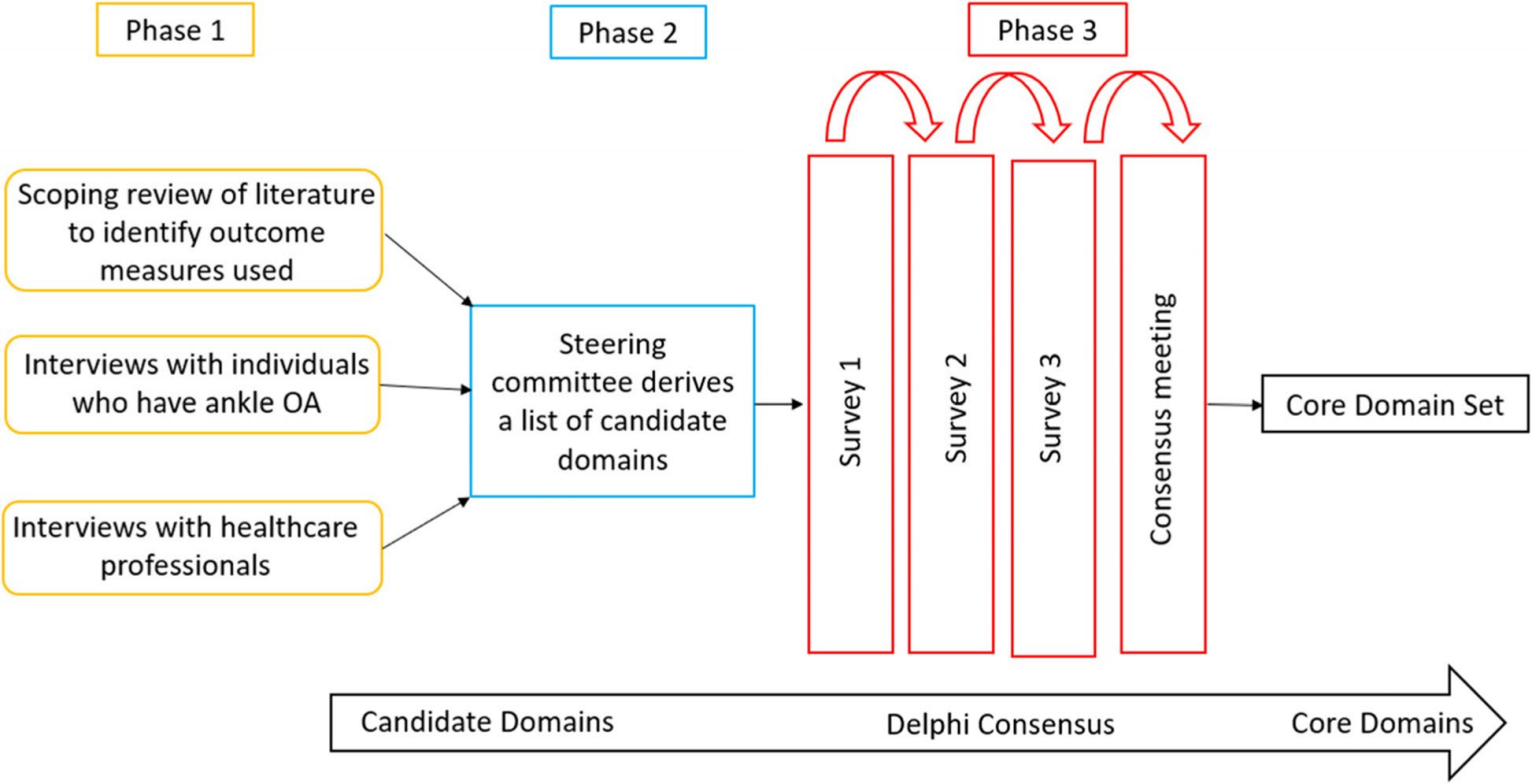
An overview of the study methods

This project will involve three phases: (1) the identification of candidate domains, (2) derivation of a list of candidate domains to be presented in a Delphi survey (in the third phase), and (3) an iterative Delphi consensus approach to arrive at an agreed core domain set (Fig. 1).

An international steering committee will guide the development of a core domain set for ankle OA. The committee members are from different healthcare professions (e.g. orthopaedic surgery, physiotherapy, podiatry), work settings (e.g. clinical, research), geographical locations (e.g. Australia, North America, Europe), and sex. The steering committee will oversee the development process and review the proposed list of domains that will be presented in the first round of a Delphi survey. The steering committee will also independently participate in the rating of candidate domains and complete the consensus process.

### Phase 1: Identification of candidate domains

The initial list of candidate domains will be gleaned by a 3-stage process. First, we will conduct a scoping review of ankle OA research to extract and record all outcome measures that have been used. The protocol of this review is registered with the International Prospective Register of Systematic Reviews (PROSPERO ID #CRD42019124546). Six databases (PubMed, Web of Science, CINAHL, Cochrane, Embase, and SPORTDiscus) will be searched, and studies will be screened by two independent reviewers. We will review the characteristics of the outcome measures extracted from all primary research on ankle OA to determine the underlying health-related ankle OA domains they represent.

Second, we will conduct a qualitative study using semi-structured interviews with individuals who have ankle OA to explore their lived experience with ankle OA and to gain a comprehensive understanding of the impact of this condition. Participants in these interviews will be asked open-ended questions about their concerns in relation to their ankle OA and how it affects them. We will also ask specific questions as recommended in the core areas of the OMERACT framework [22, 37]. The use of the OMERACT framework provides comprehensive guidance to explore participants’ perspectives about their condition and allows for a comprehensive understanding of the impact of this condition on peoples’ lives from different health-related concepts. We will recruit participants until we achieve data saturation, defined as the point at which new interview data made very little or no differences to the themes identified from data analysis [38]. Interviews will be audio-recorded, transcribed verbatim, and analysed using inductive thematic analysis [38].

Third, we will carry out semi-structured interviews with an international multidisciplinary group of healthcare professionals (e.g. orthopaedic surgeons, physical therapists, athletic trainers, and podiatrists) to gain their perspectives on key problems that they believe occur with ankle OA and outcome measures they use in patients with ankle OA. Healthcare professionals who have clinical and/or research experience in ankle OA will be invited to participate in these interviews. We will explore healthcare professionals’ perspectives about ankle OA by asking open-ended questions that will allow participants to extensively speak about their opinions. In addition, the OMERACT framework will be followed to guide our interviews to gain an in-depth understanding of healthcare professionals’ perceptions on different health-related concepts. We will use a similar process described above to collect and analyse healthcare professionals’ interviews data.

### Phase 2: Develop a list of candidate domains

The outcomes identified in the scoping review and information from the semi-structured interviews will be used by the steering committee to generate a list of candidate domains. Authors SA, BV, and MDS will develop a draft list of candidate domains. This list of candidate domains will be independently reviewed and assessed by the steering committee members. The final list of generated candidate domains to be presented to participants in the next phase (phase 3) will be considered by the steering committee at a meeting. These are recommended methods of identifying candidate domains [31, 39, 40] and have been widely applied in the development of core domains for different health conditions [34, 41–45].

### Phase 3: Delphi consensus process

An international Delphi consensus process will be used in phase 3. The Delphi method is a well-established method for achieving formal consensus [46, 47]. This method has been used to reach consensus on core domains for different health conditions such as low back pain [48], shoulder disorders [41], and childhood fractures [49]. Using an online survey permits the involvement of a large number of participants from geographically distant locations, protects the anonymity of participant responses, and avoids the effect of overly vocal or dominant individuals [27].

Candidate domains will be presented in an online survey administered using Qualtrics software (Provo, UT, USA). The participants will be asked to indicate if they agree to include the candidate domains in a core domain set. For each question, the possible answers will be ‘yes’ (meaning that the domain should be included as a core domain in ankle OA), ‘no’ (meaning the domain should not be included as a core domain in ankle OA), or ‘unsure/I do not know’. We will include a definition of what the domain is and examples of outcome measures that can be used to measure the domain. Participants will be encouraged to provide comments in open-ended text boxes to explain their responses. After all candidate domains are presented to participants, there will be an opportunity for participants to suggest new candidate domains for consideration in the subsequent survey.

In the first survey round, we will provide information about the overarching aim of the study and the Delphi process, collect demographic information (e.g. age, sex, country, level of education, profession), and seek the participant responses on the candidate domains. Frequency of responses will be calculated to determine consensus on candidate domains.

Participants who have completed the first survey round will be invited to participate in the second survey. The second survey will contain a summary report of the first survey findings and questions about domains from survey 1 that did not reach consensus (see definition below) and any new domains suggested from the open-ended responses asking for new candidate domains for consideration. The phrasing of questions about domains will be similar to that described for survey 1.

A third survey will be used to review unresolved items from survey 2, using the same format described above. If consensus has been reached for all candidate domains after the third survey, we will finalise a report of the agreed-upon core domain set. If consensus on all candidate domains has not been reached, we will arrange an online consensus meeting of survey responders to seek a final consensus. This will occur via Zoom at a time that enables maximum participation. We will send the results of the Delphi surveys to participants in advance for them to prepare for the meeting. At the meeting, we will present the undecided domains to participants, allow time for discussion about the domain, and then ask participants to independently vote. Consensus definitions will remain the same throughout, and if consensus is not reached, we will provide an explanation for the discordance.

In the event that there are a large number of domains ranked as important, we will follow the process described by OMERACT which involves placing domains into three levels of importance: (1) domains mandatory in all trials (inner circle), (2) domains that are important but optional (middle circle), or (3) research agenda domains (outer circle).

## Consensus definition

Frequency and percentage of ‘yes’, ‘no’, and ‘unsure/I do not know’ will be calculated to determine the level of agreement. We have defined consensus as ≥ 70% of participant responses by both the patient and healthcare professional groups [29]. That is, if ≥ 70% in each of the participant groups (patients and healthcare professionals) indicate that ‘yes’, the candidate domain is important enough to be included in a core domain for ankle OA, then agreement will have been reached to include the candidate domain in the core domain set. If ≥ 70% of participants answer ‘no’ to the question asking if ‘the candidate domain is important enough to be included in a core domain for ankle OA’, then agreement will have been reached to not include the candidate domain in the core domain set. Domains that do not reach consensus (≥ 70% agreement) during survey 1 will be presented to participants in survey 2 along with a summary of reasons for ‘yes’, ‘no’, and 'unsure' selections provided by participants. Reasons for selection will be analysed using the thematic analysis approach as described by Braun and Clarke [38] and will be provided as themes in the subsequent survey for each domain that did not reach a consensus. A similar process will occur for analysis of surveys 2 data and presentation of survey 3.

The final core domain set will require a consensus of both ankle OA patients and health care professionals.

## Ethical approval

We have obtained ethical approval for this project from the Institutional Human Research Ethics Committee (Approval #2019002321). All participants involved will provide their consent before participation in the study.

## Dissemination

To increase the translation, uptake, and implementation of our research, we plan to publish the final findings of the study in a peer-reviewed journal and the COMET database. We will also present our findings at national and international conferences (e.g. Osteoarthritis Research Society International, and International Ankle Consortium), patient support groups (e.g. Arthritis Australia, Musculoskeletal Australia, and Arthritis Foundation), and primary care practitioner resource repositories (e.g. Royal Australian College of General Practitioners). We will use social media platforms to disseminate and share the main study findings. A plain summary of the study results will be available to all participants.

## Discussion

The expected outcome of this project is the development of a core domain set for ankle OA that will be recommended to be used in clinical trials of ankle OA. This is an important first step to developing a core outcome set for ankle OA. The development of the core domain set will involve relevant stakeholder groups (e.g. patients, healthcare professionals) to ensure that key perspectives are included.

This project follows the current recommended guidelines for the development of a high-quality core domain set [22, 31, 50]. As we have undertaken to include different methodological approaches to collect a wide range of candidate domains, there may be methodological challenges that need to be considered. A scoping review of the current outcome measures used in ankle OA clinical studies will be conducted and candidate domains based on these outcome measures will be proposed. Although we will aim to broadly search in the most relevant electronic databases, there is a possibility that some articles may not be identified. Further, we will only include articles in the English language in this review, which may lead to language or cultural bias.

Another source of candidate domains will be semi-structured interviews with two important stakeholder groups—persons who live with ankle OA and healthcare professionals. These interviews will be limited to individuals who have sufficient English language proficiency. The restriction of English language eligibility is an inevitable limitation to allow clear communication between the research team and participants.

Key stakeholder involvement in the consensus process is an integral part of this process. There is a lack of guidance on how many stakeholders are recommended in total, or on the size and composition of groups, but there is anticipation that these factors may influence what domains are ranked as important [31, 50, 51]. While there is no consensus on the optimum number of Delphi participants, it is recommended that the number of participants from each stakeholder group consists of 15 to 30 participants [47]. In this project, participants will include individuals who have ankle OA as well as an international multidisciplinary group of healthcare professionals. It is possible that the group representatives maybe not be equally distributed. We will attempt to include a similar number of stakeholders using the different recruitment methods described above.

Once a core domain set is developed for ankle OA, the first step in core outcome set development, future research will be needed to develop a core set of outcome measurement instruments (i.e. core outcome set) that corresponds to the agreed-upon core domains for ankle OA. The development of a core outcome set will be a separate research activity that requires another protocol.

## Conclusion

In this protocol paper, we describe the methods that will be used to develop a core domain set for ankle OA. These methods are based on the current recommendations and guidelines of international initiatives to develop a health-related core domain set. Individuals with ankle OA and healthcare professionals will be included in the process, ensuring that relevant stakeholder perspectives are captured. The overarching aim of this project is to develop an internationally agreed-upon set of core domains for ankle OA. This is a fundamental step toward the future development of a core outcome set for ankle OA. A core outcome set for ankle OA is expected to increase the reporting of important outcomes, minimise the risk of selective outcome reporting bias, and facilitate future systematic reviews and meta-analyses, which are considered the highest level of evidence-based practice. This process will progress evidence for the management of ankle OA.

## Project status

The project is ethically approved by the Institutional Human Research Ethics Committee (approval #2019002321) and is registered with the COMET initiative database. The protocol of the scoping review that is part of this project is registered with the PROSPERO database (ID #CRD42019124546).

## Supplementary Information

**Additional file 1.** Core Outcome Set-STAndardised Protocol Items (COS-STAP) Checklist

## Data Availability

We have registered this protocol with the COMET initiative database. A full report of the output of the research proposed in this protocol will be submitted for publication in a peer-review journal. All relevant data will be available on request from the authors.

## Abbreviations

OA: Osteoarthritis
OMERACT: Outcome Measures in Rheumatology
COMET: Core Outcome Measures in Effectiveness Trials
COS-STAP: Core Outcome Set-STAndardised Protocol Items
