# Supplementary material for: Protocol for the development of a core domain set for individuals with ankle osteoarthritis
Source: Trials. 2022 Sep 5;23:748. doi: 10.1186/s13063-022-06692-0 (PMC9446870; doi:10.1186/s13063-022-06692-0)
Supplement: Supplementary file 1 — Additional file 1. Core Outcome Set-STAndardised Protocol Items (COS-STAP) Checklist [file 13063_2022_6692_MOESM1_ESM.pdf]

Additional file 1

Core Outcome Set-STAndardised Protocol Items (COS-STAP) Checklist

Checklist Item	Item No	Checklist Item Details	Page number
**TITLE/ABSTRACT**			
Title	1a	Identify in the title that the paper describes the protocol for the planned development of a COS	1
Abstract	1b	Provide a structured abstract	2
INTRODUCTION			
Background and objectives	2a	Describe the background and explain the rationale for developing the COS, and identify the reasons why a COS is needed and the potential barriers to its implementation	3
	2b	Describe the specific objectives with reference to developing a COS	3
Scope	3a	Describe the health condition(s) and population(s) that will be covered by the COS	4
	3b	Describe the intervention(s) that will be covered by the COS	4
	3c	Describe the context of use for which the COS is to be applied	4
METHODS			
Stakeholders	4	Describe the stakeholder groups to be involved in the COS development process, the nature of and rationale for their involvement and also how the individuals will be identified; this should cover involvement both as members of the research team and as participants in the study	4&5
Information sources	5a	Describe the information sources that will be used to identify the list of outcomes. Outline the methods or reference other protocols/papers	5&6
	5b	Describe how outcomes may be dropped/combined, with reasons	6&7
Consensus process	6	Describe the plans for how the consensus process will be undertaken	5, 6&7
Consensus definition	7a	Describe the consensus definition	7
	7b	Describe the procedure for determining how outcomes will be added/combined/dropped from consideration during the consensus process	6&7
ANALYSIS			
Outcome scoring/feedback	8	Describe how outcomes will be scored and summarised, describe how participants will receive feedback during the consensus process	6&7
Missing data	9	Describe how missing data will be handled during the consensus process	6&7
ETHICS and DISSEMINATION			
Ethics approval/informed consent	10	Describe any plans for obtaining research ethics committee/institutional review board approval in relation to the consensus process and describe how informed consent will be obtained (if relevant)	7&8

Additional file 1

Dissemination	11	Describe any plans to communicate the results to study participants and COS users, inclusive of methods and timing of dissemination	8
ADMINISTRATIVE INFORMATION			
Funders	12	Describe sources of funding, role of funders	10
Conflicts of interest	13	Describe any potential conflicts of interest within the study team and how they will be managed	10
